# Unilateral vocal cord paralysis following arthroscopic rotator cuff repair in the beach chair position: a case series

**DOI:** 10.1097/RC9.0000000000000947

**Published:** 2026-09-08

**Authors:** Giyong Yoon, Jiwon Bak, Minsoo Kim

**Affiliations:** Department of Anesthesiology and Pain Medicine, Kangwon National University Hospital, Kangwon National University College of Medicine, Chuncheon, South Korea

**Keywords:** beach chair position, case series, hypotension, recurrent laryngeal nerve, shoulder arthroscopy, vocal cord paralysis

## Abstract

**Introduction:**

Vocal cord paralysis (VCP) is a rare complication of endotracheal intubation. During shoulder surgery in the beach chair position, deliberate hypotension may compromise perfusion of laryngeal structures. This case series was conducted to describe the clinical characteristics of postoperative VCP in this setting and to explore the potential role of intraoperative hypotension as an underlying mechanism.

**Case presentations:**

This retrospective single-center case series included four consecutive female patients with hypertension who developed unilateral (left-sided) VCP after arthroscopic rotator cuff repair in the beach chair position under general anesthesia. Intraoperatively, mean blood pressure was maintained at low levels, with periods below 60 mmHg in all cases. Postoperatively, patients presented with hoarseness and aspiration. Laryngoscopic examination confirmed left-sided VCP in all cases, and laryngeal electromyography demonstrated recurrent laryngeal nerve neuropathy in two cases. All patients improved with conservative management, and no statistical analysis was performed given the descriptive nature of this case series.

**Discussion:**

Direct airway trauma, endotracheal tube malposition, and cuff overinflation were unlikely causes. Instead, reduced perfusion pressure related to head elevation and intraoperative hypotension may have contributed to ischemic injury of the recurrent laryngeal nerve. These findings suggest that postoperative VCP may involve factors beyond traditional mechanical airway-related mechanisms and warrant further investigation of perioperative hemodynamic influences.

**Conclusion:**

Shoulder surgery in the beach chair position combined with intraoperative hypotension may predispose patients to postoperative VCP. This case series extends previous observations by highlighting a potential association between intraoperative hemodynamic management and postoperative recurrent laryngeal nerve dysfunction. These findings underscore the potential importance of individualized intraoperative blood pressure management in reducing the risk of this complication. Further prospective studies are warranted to clarify the relationship between head elevation, regional perfusion, and laryngeal nerve injury during shoulder surgery.

## Introduction

Endotracheal intubation in patients with a normal airway is generally considered a safe procedure during general anesthesia. Nevertheless, a wide spectrum of complications may occur, ranging from postoperative sore throat (the most common) to vocal cord paralysis (VCP), a rare but potentially serious complication with a reported incidence of less than 0.1%^[^1,2^]^.

HIGHLIGHTS

- Unilateral vocal cord paralysis can occur after shoulder surgery in the beach-chair position.
- Sustained intraoperative hypotension may contribute to ischemic recurrent laryngeal nerve injury.
- All cases demonstrated gradual recovery with conservative management, suggesting a potentially reversible injury.
- Attention to intraoperative hemodynamic management may reduce the risk of this complication.

VCP can result in significant adverse outcomes such as aspiration pneumonia, thereby increasing postoperative morbidity and mortality. Therefore, early identification of the underlying cause is essential for prompt management. Neurogenic VCP may arise from injury to the vagus nerve or recurrent laryngeal nerve (RLN) due to surgical manipulation, neoplastic invasion, or endotracheal tube (ETT)-related factors. Mechanical fixation of the vocal folds or arytenoid cartilage can also occur due to direct laryngeal trauma. Systemic conditions, including lung cancer and cerebrovascular disease, have also been implicated in the development of VCP^[^3^]^.

Patient positioning during surgery may lead to the development of VCP. Lim *et al* reported on 43 patients with VCP following general anesthesia with endotracheal intubation, among whom upper extremity surgery was the most frequent surgical category (12 cases), and 75% (9 cases) were performed in the sitting position^[^1^]^. Olson *et al*^[^4^]^ and Park *et al*^[^5^]^ each reported a case of VCP following arthroscopic rotator cuff repair (RCR) performed in a beach chair position (BCP) and suggested that the BCP itself might be a contributing factor to the occurrence of VCP. While previous reports have suggested a positional association between the BCP and postoperative VCP, they were limited to isolated case reports and did not specifically examine the potential contribution of intraoperative blood pressure management. In contrast, the present case series explores the potential contribution of intraoperative hemodynamic factors and proposes a possible ischemic mechanism related to reduced laryngeal perfusion.

In this report, we present four cases of unilateral VCP following arthroscopic RCR in the BCP. Our goal is to describe the clinical characteristics of these cases and to highlight potential anesthetic and hemodynamic factors associated with this complication while emphasizing the clinical implications of intraoperative hypotension. This case series has been reported in line with the PROCESS 2025 guidelines^[^6^]^.

## Case presentations

Ethical approval for this retrospective case series was provided by the Institutional Review Board of Kangwon National University Hospital, Chuncheon, Republic of Korea (IRB No. 2026-01-004) on 26 January 2026. The requirement for written informed consent was waived because this study involved a retrospective review of anonymized medical record data and posed minimal risk to patients.

This retrospective single-center case series included four consecutive adult female patients who developed new-onset unilateral VCP after elective arthroscopic RCR in the BCP. All patients were classified as American Society of Anesthesiologists (ASA) physical status II and had hypertension, with or without diabetes mellitus. Inclusion criteria included adult patients with postoperative unilateral VCP after shoulder arthroscopy in the BCP, while patients with pre-existing laryngeal disease; prior neck or laryngeal surgery with potential involvement of the RLN; malignancy involving the neck; or documented traumatic intubation were excluded.

Patient data were retrospectively identified through electronic medical records and otolaryngology consultation records and fully anonymized prior to analysis. All procedures were elective and conducted according to routine institutional practice. Arthroscopic RCR was performed under general anesthesia in the BCP, with deliberate intraoperative hypotension used to optimize surgical visualization. Standard anesthetic techniques for induction, airway management, patient positioning, intraoperative monitoring, and cuff pressure control were applied across all cases, and no novel surgical or anesthetic devices were used.

Postoperative evaluation was conducted in collaboration with otolaryngology specialists, using clinical assessment and fiber-optic or stroboscopic laryngoscopy, with adjunctive imaging or laryngeal electromyography (EMG) when indicated. Follow-up protocols were standardized across cases and were performed in the outpatient setting for up to 3 months. Data were analyzed descriptively using a narrative approach without formal statistical testing. This retrospective case series is reported in accordance with the PROCESS 2025 guidelines^[^6^]^.

## Case 1

A 49-year-old woman (height, 151 cm; weight, 61.3 kg) with a right rotator cuff tear was scheduled for arthroscopic RCR. Her medical history was notable only for hypertension. The results of preoperative laboratory investigations and airway assessments were unremarkable.

Standard monitoring, including electrocardiography, pulse oximetry, non-invasive blood pressure measurement, and neuromuscular transmission monitoring, was performed. After pre-oxygenation with 100% oxygen for 3 minutes, general anesthesia was induced with propofol (120 mg). After confirming adequate mask ventilation, rocuronium (40 mg) and esmolol (30 mg) were administered. Orotracheal intubation was performed uneventfully with a 7.0-mm internal-diameter (ID) ETT (Mallinckrodt, Covidien, USA) under direct laryngoscopy when the train-of-four count reached zero. The ETT was secured at 21 cm at the left corner of the mouth, and the cuff was inflated with 4 mL of air. The cuff pressure was maintained between 20 and 30 cm H_2_O using a handheld cuff-pressure gauge (Portex, Smiths Medical, USA).

Anesthesia was maintained with oxygen, medical air, and sevoflurane (2.0–2.5 vol%) without nitrous oxide (N_2_O). The patient was placed in a BCP at an elevation of approximately 70°, which was maintained throughout the procedure. The mean blood pressure (MBP) ranged from 55 to 75 mmHg; however, despite adjustments to anesthetic depth and intermittent vasopressor administration, the MBP remained below 60 mmHg for approximately 30 min. The surgical and anesthetic durations were 205 and 260 min, respectively. Extubation was uneventful, and no blood was observed on the ETT or suction apparatus.

On postoperative day 1, the patient complained of hoarseness but denied having a sore throat, aspiration symptoms, or neck discomfort. Laryngeal stroboscopy, performed by an otolaryngologist, revealed fixation of the left vocal fold in the paramedian position (Fig. 1). One month later, laryngeal EMG revealed left recurrent laryngeal neuropathy. After approximately 3 months of conservative management, the patient’s hoarseness resolved, and the mobility of the left vocal fold was restored.

**Figure 1.**
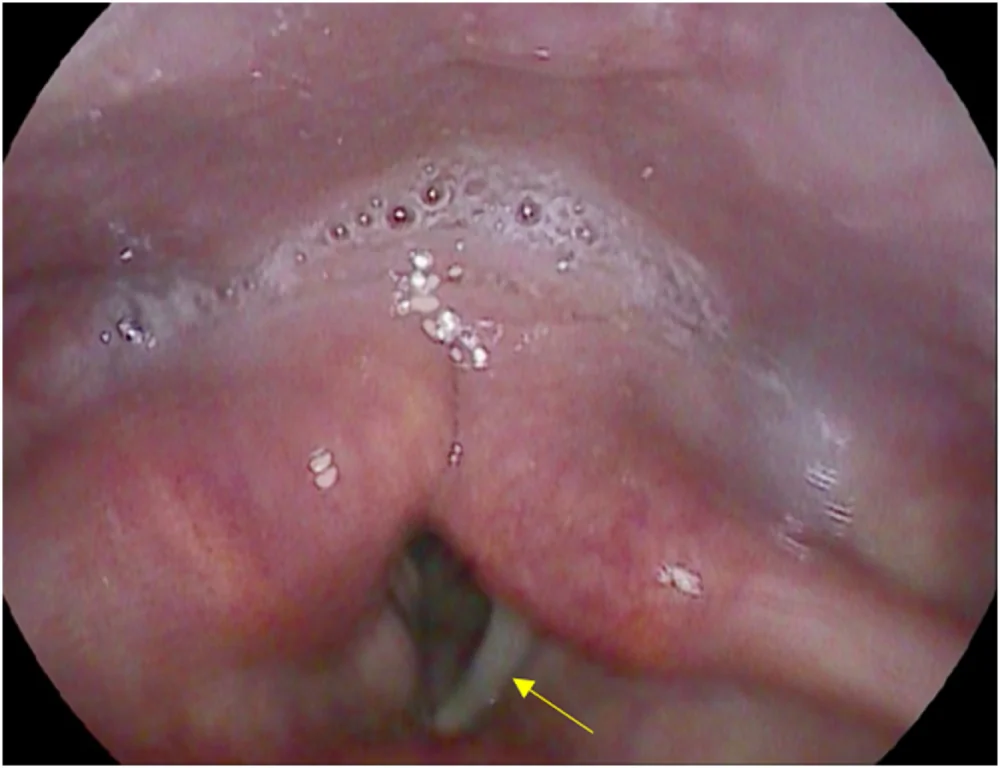
Stroboscopic image of the vocal folds demonstrating left-sided VCP. The arrow indicates fixation of the left vocal fold in the paramedian position.

## Case 2

A 60-year-old woman with hypertension underwent arthroscopic repair of a right rotator cuff tear. Airway management was uneventful, as in Case 1. Surgery was performed in the BCP. Intraoperatively, MBP ranged from 55 to 70 mmHg, with intermittent decreases below 60 mmHg for a cumulative duration of approximately 20 minutes.

Postoperatively, the patient experienced hoarseness, sore throat, and aspiration lasting approximately 5 days. Neck computed tomography revealed no structural laryngeal lesions other than asymmetry of the left true vocal fold (Fig. 2). Laryngeal EMG performed 1 month later demonstrated left recurrent laryngeal neuropathy, and the symptoms gradually improved with conservative management.

**Figure 2.**
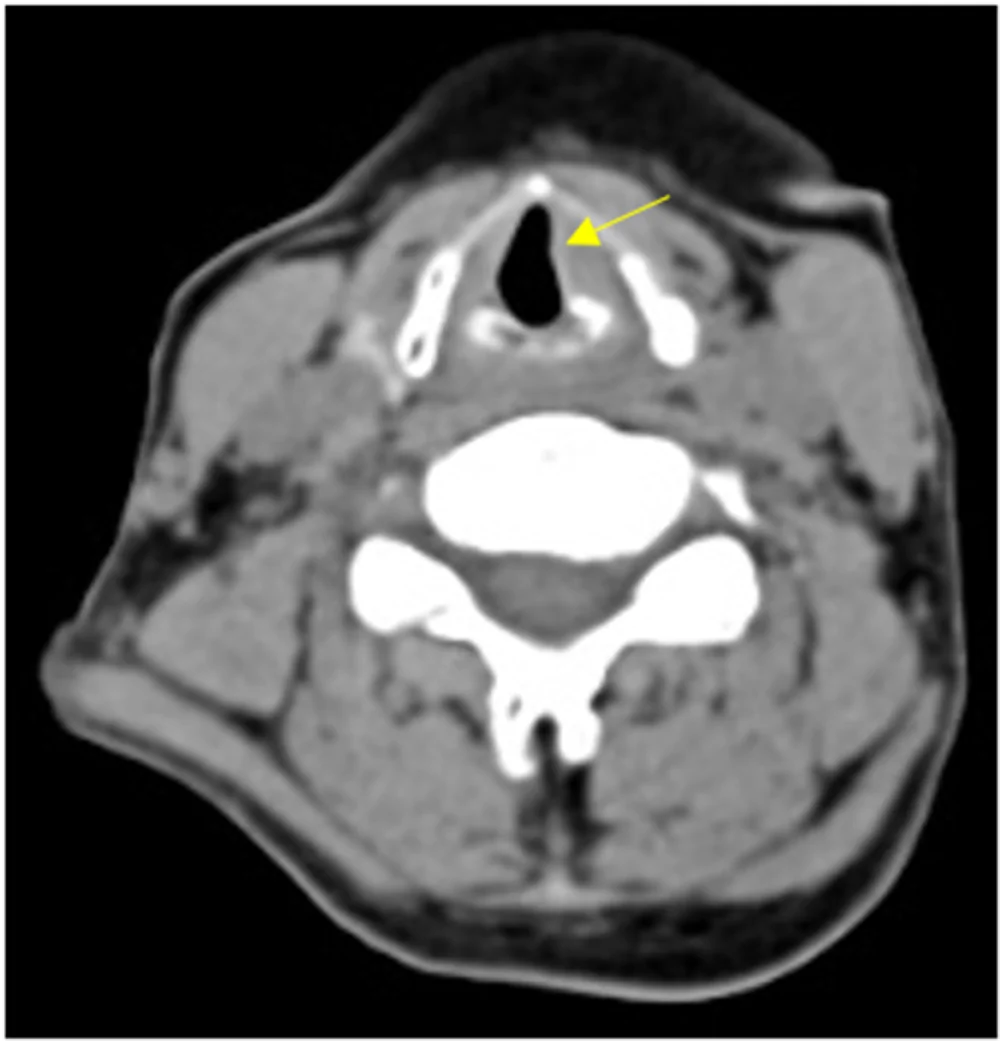
Neck computed tomography image obtained on postoperative day 5. The arrow indicates fixation of the left vocal fold in the paramedian position.

## Case 3

A 67-year-old woman with hypertension and diabetes mellitus underwent arthroscopic repair of a left rotator cuff tear. Airway management and anesthetic techniques were similar to those in case 1. Surgery was performed in the BCP, and intraoperative MBP ranged from 50 to 75 mmHg.

On postoperative day 2, the patient complained of hoarseness and aspiration. Fiberoptic laryngoscopy revealed fixation of the left vocal fold in a paramedian position with hypomobility. Although laryngeal EMG was recommended, the patient declined further evaluation because her symptoms improved during follow-up.

## Case 4

A 59-year-old woman with hypertension, diabetes mellitus, and a history of cervical spine surgery underwent arthroscopic repair of a right rotator cuff tear. Tracheal intubation using video laryngoscopy was uneventful. Surgery was performed in the BCP, with intraoperative MBP maintained between 50 and 70 mmHg.

Postoperatively, the patient experienced hoarseness and aspiration lasting for approximately 3 weeks. Fiber-optic laryngoscopy demonstrated immobility of the left vocal fold. As symptoms gradually improved, additional diagnostic studies were not pursued, and follow-up laryngoscopy demonstrated marked improvement in vocal fold mobility.

Clinical characteristics and outcomes of the four patients are summarized in Table 1. Postoperative unilateral VCP was evaluated by otolaryngology using fiberoptic laryngoscopy and/or stroboscopic assessment, with adjunctive neck computed tomography and laryngeal EMG when indicated. All patients were managed conservatively with observation and outpatient follow-up, experienced gradual symptomatic and functional recovery, and had no adverse events. No deviations from the planned perioperative management were observed across cases.

**Table 1:** Clinical characteristics, diagnostic evaluations, and outcomes of the four patients with postoperative unilateral vocal cord paralysis following arthroscopic rotator cuff repair in the beach chair position.

Case No.	Preoperative condition	Intraoperative event (beach chair position)	Early postoperative findings	Follow-up evaluation	Final outcome (recovery)
Case 1	49/F, HTN	MBP < 60 mmHg (30 min)	Hoarseness and left VCP (stroboscopy)	Left RLN neuropathy (EMG)	Full recovery at 3 months
Case 2	60/F, HTN	MBP < mmHg (20 min)	Hoarseness, sore throat, and aspiration	Left RLN neuropathy (EMG)	Gradual improvement
Case 3	67/F, HTN, DM	MBP 50–75 mmHg (low range)	Hoarseness, aspiration, and left vocal fold hypomobility	Improved symptoms (refused EMG)	Gradual improvement
Case 4	59/F, HTN, DM, C-spine surgery	MBP 50–70 mmHg (low range)	Hoarseness, aspiration, and left VCP	Improved mobility (laryngoscopy)	Marked improvement

DM, diabetes mellitus; EMG, electromyography; HTN, hypertension; MBP, mean blood pressure; RLN, recurrent laryngeal nerve; VCP, vocal cord paralysis.

## Discussion

In this case series, left-sided VCP occurred after arthroscopic RCR in the BCP in the setting of intraoperative hypotension. Importantly, this complication developed despite the absence of recognized mechanical risk factors for laryngeal nerve injury, including difficult intubation, excessive cuff pressure, or direct surgical manipulation. These observations prompted further consideration of non-mechanical mechanisms contributing to postoperative VCP in this surgical context.

Airway injuries associated with endotracheal intubation are well-known complications of general anesthesia. According to the ASA Closed Claims Database, the larynx is the most frequently injured site, accounting for approximately 33% of all airway-related injuries^[^7^]^. Laryngeal complications associated with endotracheal intubation encompass a range of conditions, including VCP, granuloma formation, arytenoid dislocation, submucosal hemorrhage, hematoma, and subglottic edema. Among these complications, VCP is the most frequently reported anesthesia-related laryngeal injury, followed by granuloma formation and arytenoid cartilage displacement^[^7^]^.

Arytenoid dislocation can produce clinical symptoms that mimic VCP and is typically associated with traumatic intubation during difficult airway management under general anesthesia^[^8^]^. Left-sided arytenoid dislocations are more frequent, possibly because ETTs are usually inserted from the right side of the oral cavity and secured at the right angle of the mouth^[^2^]^. In the present case series, tracheal intubation was performed smoothly in all patients, without evidence of traumatic insertion. Although one patient (case 4) had a history of prior cervical spine surgery and was anticipated to have a potentially difficult airway, tracheal intubation was successfully achieved using GlideScope^®^ video laryngoscopy without difficulty, making arytenoid dislocation unlikely in this case.

RLN injury is a recognized cause of VCP and may result from direct surgical trauma, traction-related nerve injury, or ischemic and compressive mechanisms^[^9–11^]^. The surgical procedures most commonly associated with RLN injury include thyroid, anterior cervical spine, thoracic, and cardiac surgeries^[^1,12^]^. Because the procedures in the present cases were limited to arthroscopic shoulder surgery, direct surgical manipulation of the RLN was unlikely. Arthroscopic shoulder surgery requires continuous irrigation to maintain a clear operative field, which can result in regional soft-tissue edema and may exert compressive effects on the laryngeal and cervical tissues adjacent to the shoulder. Although this mechanism might be a contributing factor, all four patients developed left-sided VCP regardless of the side of surgery, suggesting that local surgical factors were unlikely to be the primary cause. However, in one case (case 3) involving left shoulder surgery followed by left-sided VCP, unilateral regional edema or compressive effects related to surgical positioning or irrigation could not be entirely excluded as contributing mechanisms.

The ETT cuff pressure is an important factor in the prevention of laryngeal and tracheal complications. Excessive cuff pressure may compromise tracheal mucosal perfusion and cause ischemic injury to adjacent neural structures, including the RLN^[^13,14^]^. Several factors can influence cuff pressure, including patient positioning, head and neck movement, temperature changes, and the use of N_2_O^[^15–18^]^. Maintaining cuff pressure between 20 and 30 cm H_2_O to preserve mucosal blood flow is recommended^[^19^]^. In the present cases, cuff pressure was measured immediately after intubation using a cuff pressure gauge and was maintained within the recommended range throughout anesthesia. N_2_O, which can increase cuff pressure by diffusing into the cuff, was not used in any case, making cuff over-inflation unlikely. An improper ETT insertion depth may also contribute to RLN injury. When the tube is positioned too shallowly, the cuff may be inflated within the larynx, compressing the anterior branch of the RLN between the cuff and thyroid cartilage^[^20^]^. Optimal insertion depth for oral intubation is generally 20–21 cm in women and 22–23 cm in men^[^21^]^. Although all patients were shorter than the average height of Korean women, the ETT was secured at 21 cm in all cases, and the cuff pressure was maintained within the recommended range, making clinically significant ETT malposition unlikely.

Rather than direct airway trauma or focal RLN injury, intraoperative hypotension associated with the BCP may compromise the microcirculation to the laryngeal structures. Owing to hydrostatic effects, arterial pressure decreases with increasing vertical height above the blood pressure cuff at an estimated rate of approximately 0.77 mmHg per centimeter^[^22^]^. In this position, the 15–20 cm vertical distance between the heart and vocal cords can decrease mean arterial pressure at the laryngeal level by approximately 11–15 mmHg. During arthroscopic shoulder surgery, surgeons commonly request deliberate hypotension to optimize the surgical field. In cases 1 and 2, MBP decreased to less than 60 mmHg for approximately 30 and 20 minutes, respectively. In cases 3 and 4, MBP was generally maintained within a low range (50–75 mmHg and 50–70 mmHg, respectively), with the lowest recorded values of approximately 50 mmHg. However, the cumulative duration of MBP less than 60 mmHg could not be quantified from the available records. Such sustained or repetitive reductions in perfusion pressure may have led to impaired microcirculation and ischemic injury to the RLN. This effect may have been further amplified by pre-existing hypertension, which can increase susceptibility to neural ischemia during episodes of systemic hypotension. In addition, the left RLN is anatomically longer than the right and follows a more complex intrathoracic course, potentially rendering it more vulnerable to ischemic insults^[^13^]^. These factors may explain the consistent left-sided involvement observed in all cases.

Over the past 5 years at our institution, postoperative VCP occurred in only six cases out of approximately 20 000 general anesthesia procedures. While two cases were attributable to difficult intubation and presumed direct trauma, the remaining four cases were observed exclusively after shoulder surgery performed in the BCP. Although this observation does not establish causality, the consistent institutional pattern observed under similar anesthetic and surgical conditions, together with the systematic exclusion of alternative etiologies, provides additional support for the proposed hypothesis that head elevation and intraoperative hypotension may contribute to the development of postoperative VCP.

Although the proposed ischemic mechanism is biologically plausible, the present case series cannot establish a causal relationship between intraoperative hypotension and RLN injury. Therefore, this hypothesis should be considered exploratory and hypothesis-generating. The principal limitations of this study are the small sample size and the retrospective design without a control group, which substantially limit generalizability and preclude definitive causal inference. Laryngeal EMG was not performed in all cases; neuropathy was electrophysiologically confirmed in two patients, whereas the diagnoses in the remaining cases were based on clinical and laryngoscopic findings alone. Additionally, blood pressure was measured in the arm rather than at the level of the vocal cords, and detailed data on regional laryngeal perfusion were unavailable. Pre-existing subclinical laryngeal nerve dysfunction could not be completely excluded because routine preoperative laryngoscopic evaluation was not performed. There were no deviations from the planned perioperative anesthetic or surgical management across cases, as all patients underwent routine arthroscopic shoulder surgery under standard general anesthesia protocols. In addition, this case series did not involve any novel anesthetic techniques or medical devices, and therefore, specific contraindications or device-related risks beyond established perioperative considerations could not be assessed. These limitations should be considered when interpreting the findings and extrapolating them to broader patient populations.

Current perioperative airway management standards primarily focus on preventing mechanical causes of VCP, such as traumatic intubation, ETT malposition, and excessive cuff pressure. Our findings suggest that, in the context of shoulder surgery performed in the BCP, adherence to these airway-related standards alone may be insufficient. Instead, anesthetic management strategies should also account for regional perfusion pressure, particularly when deliberate hypotension is employed. This observation generates the hypothesis that head elevation-related hydrostatic gradients combined with systemic hypotension may represent an under-recognized mechanism of RLN ischemia, warranting further investigation and consideration in future perioperative hemodynamic guidelines. Although definitive blood pressure thresholds cannot be derived from this small case series, avoiding prolonged periods of MBP below 60 mmHg and considering the hydrostatic gradient between the arm and head may represent reasonable interim strategies, particularly in patients with pre-existing hypertension undergoing shoulder surgery in the BCP.

This case series raises several important questions for future research. In particular, the optimal intraoperative blood pressure threshold and duration of hypotension that can be safely tolerated during shoulder surgery in the BCP remain unclear, especially in patients with pre-existing hypertension. Future studies should also explore whether regional perfusion pressure at the level of the head and neck more accurately predicts neurologic complications than conventional arm-based blood pressure measurements. Prospective observational studies with standardized hemodynamic protocols, as well as controlled comparative studies evaluating different blood pressure targets, would be best suited to clarify these relationships and to inform evidence-based perioperative hemodynamic guidelines.

All patients were managed conservatively, without additional interventions or prolonged hospitalization, and the avoidance of excessive intraoperative hypotension represents a cost-neutral preventive strategy.

## Conclusion

These observations suggest a potential association between head elevation, intraoperative hypotension, and postoperative unilateral VCP. Alternative etiologies were systematically evaluated and largely excluded, and the proposed ischemic mechanism is biologically plausible. Arthroscopic RCR is commonly performed in the BCP and frequently involves deliberate hypotension to improve surgical visualization. In this retrospective case series, the combination of head elevation and sustained intraoperative hypotension was associated with the development of postoperative unilateral VCP, presumably through reduced perfusion pressure at the level of the laryngeal nerves.

Although causality cannot be established, these findings suggest that excessive or prolonged hypotension, particularly in patients with pre-existing hypertension, may increase the risk of ischemic RLN injury. From a clinical perspective, anesthesiologists should individualize intraoperative blood pressure targets and avoid unnecessary hypotension to preserve adequate head and neck perfusion during shoulder surgery in the BCP. This case series highlights the need to define safe intraoperative blood pressure thresholds in this setting, and future prospective studies incorporating standardized blood pressure targets and head-level perfusion assessments are warranted.

## Data Availability

The datasets used and/or analyzed during the current study are not publicly available because of patient confidentiality but are available from the corresponding author upon reasonable request.
